# Supplementary material for: Functional Comparison of Three Chitinases from Symbiotic Bacteria of Entomopathogenic Nematodes
Source: Toxins (Basel). 2024 Jan 3;16(1):26. doi: 10.3390/toxins16010026 (PMC10821219; doi:10.3390/toxins16010026)
Supplement: Supplementary file 1 [file toxins-16-00026-s001.zip › toxins-2755928-supplementary.pdf]

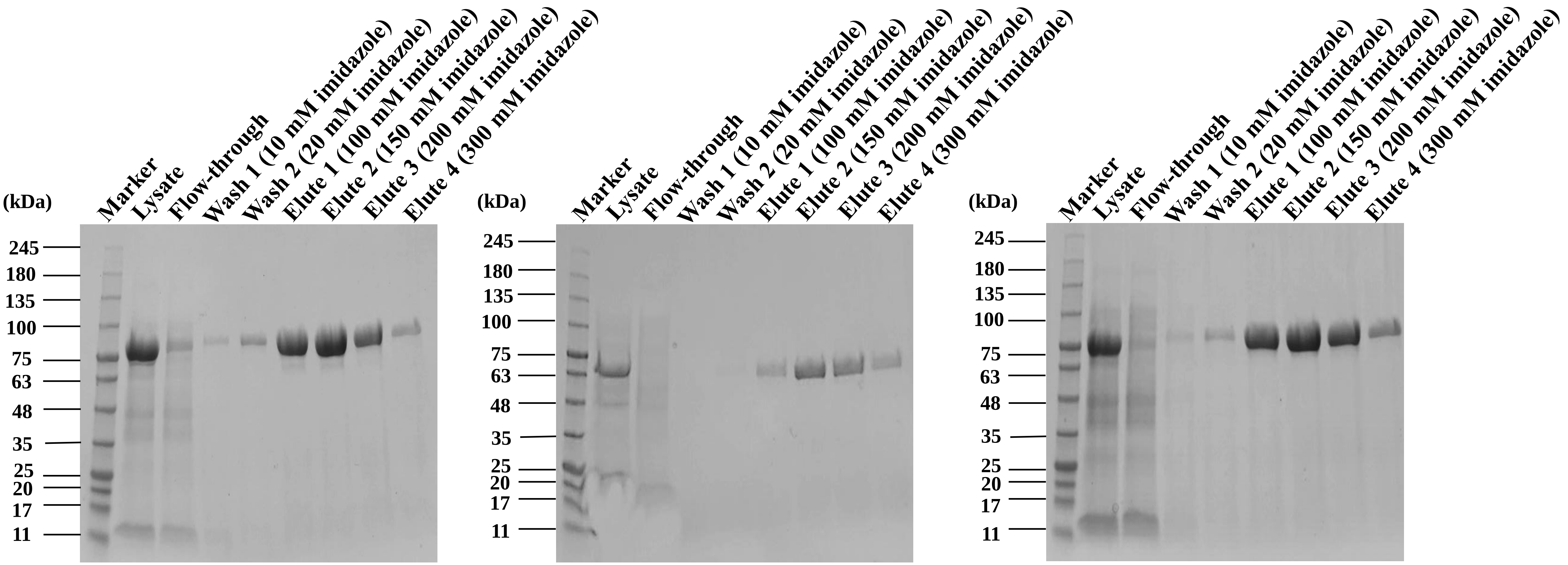

Figure S1. Cell lysate, a soluble fraction containing 6xHis-tagged chitinase protein, was purified using an imidazole concentration-dependent method using Ni-NTA superflow resin. Purified chitinase protein were analyzed by 4−15% SDS−PAGE, respectively. From the left, XnChi, XhChi, and PtChi are shown.

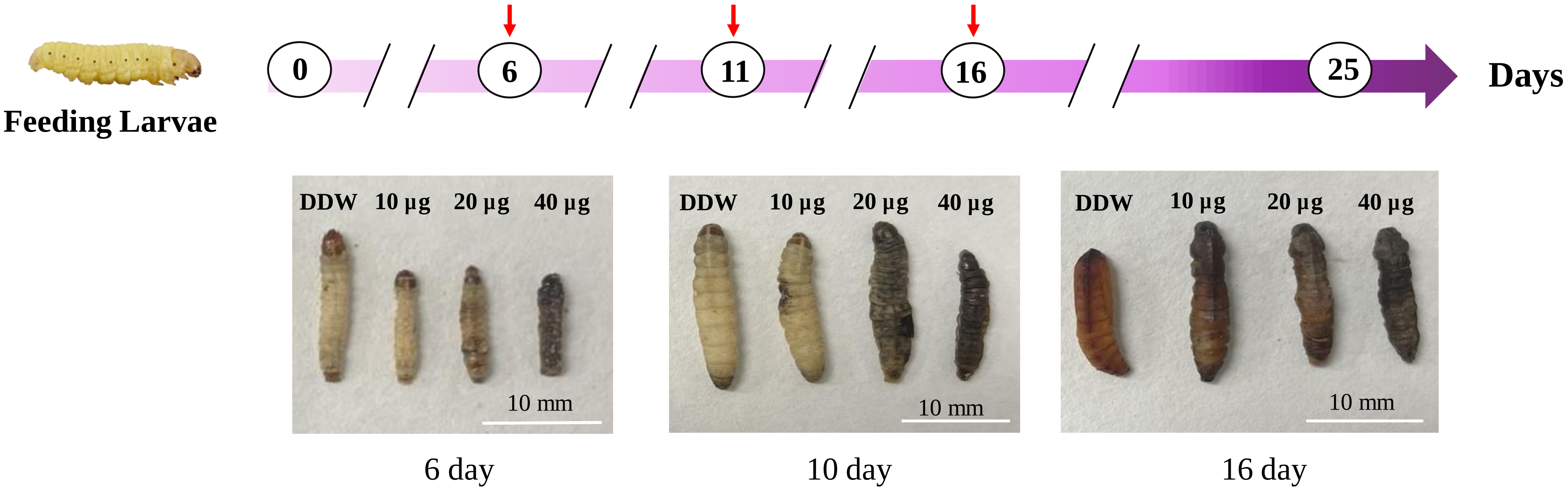

Figure S2. Oral toxicity of XnChi, XhChi, and PtChi against *G. mellonella* was observed on 25 days after treatment. The photograph represents XnChi and the arrow indicates the date of photograph taken. White bar indicates the size of larva and larval length was corrected through the image J program.

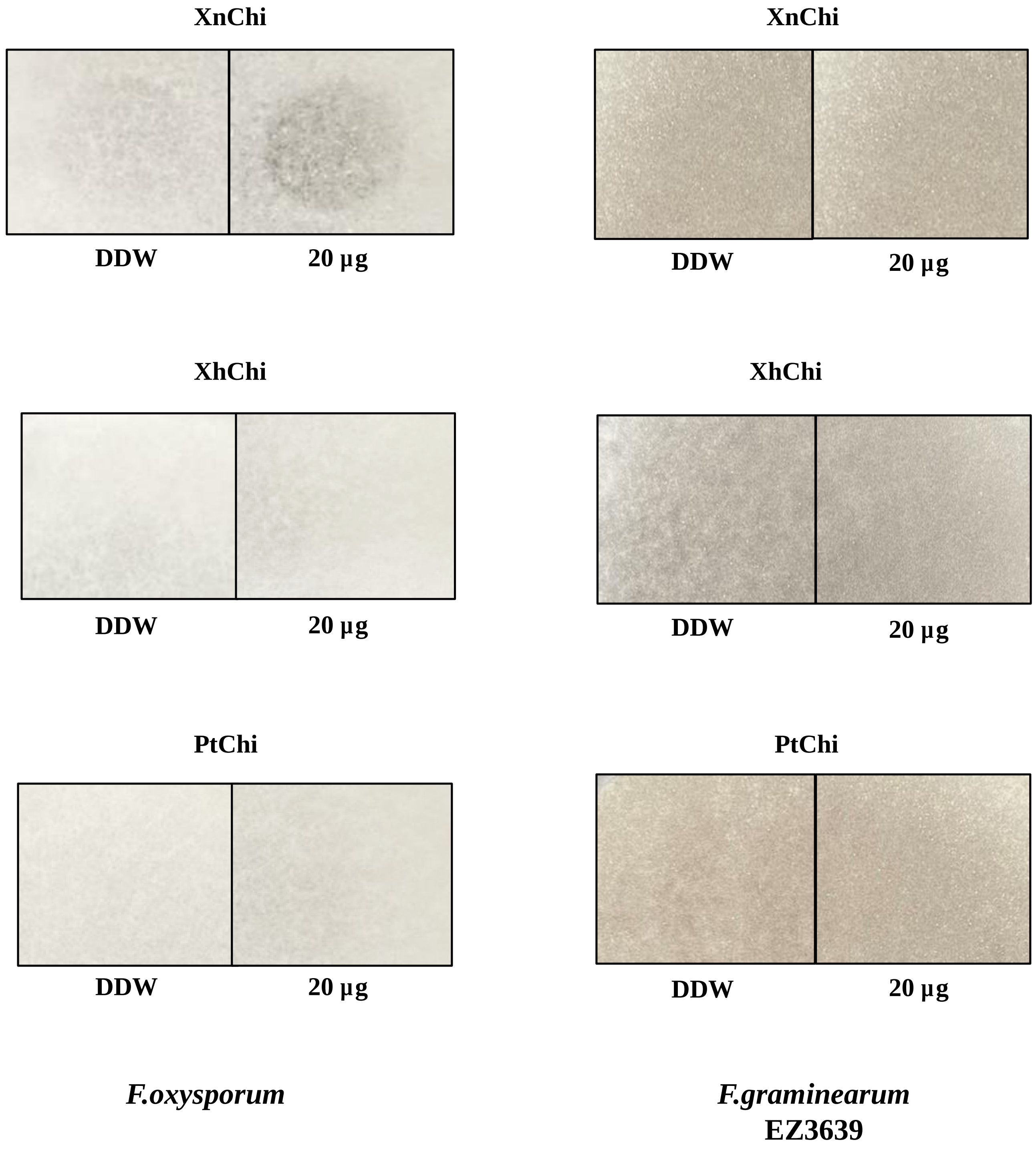

Figure S3. Antifungal activity of XnChi, XhChi, and PtChi (20 µg) against *F. oxysporum* and *F. graminearum*.
Inhibition of mycelial growth by chitinases was shown as a inhibition zone by disc diffusion susceptibility assay. As a control, DDW was used, and no growth inhibition was observed.

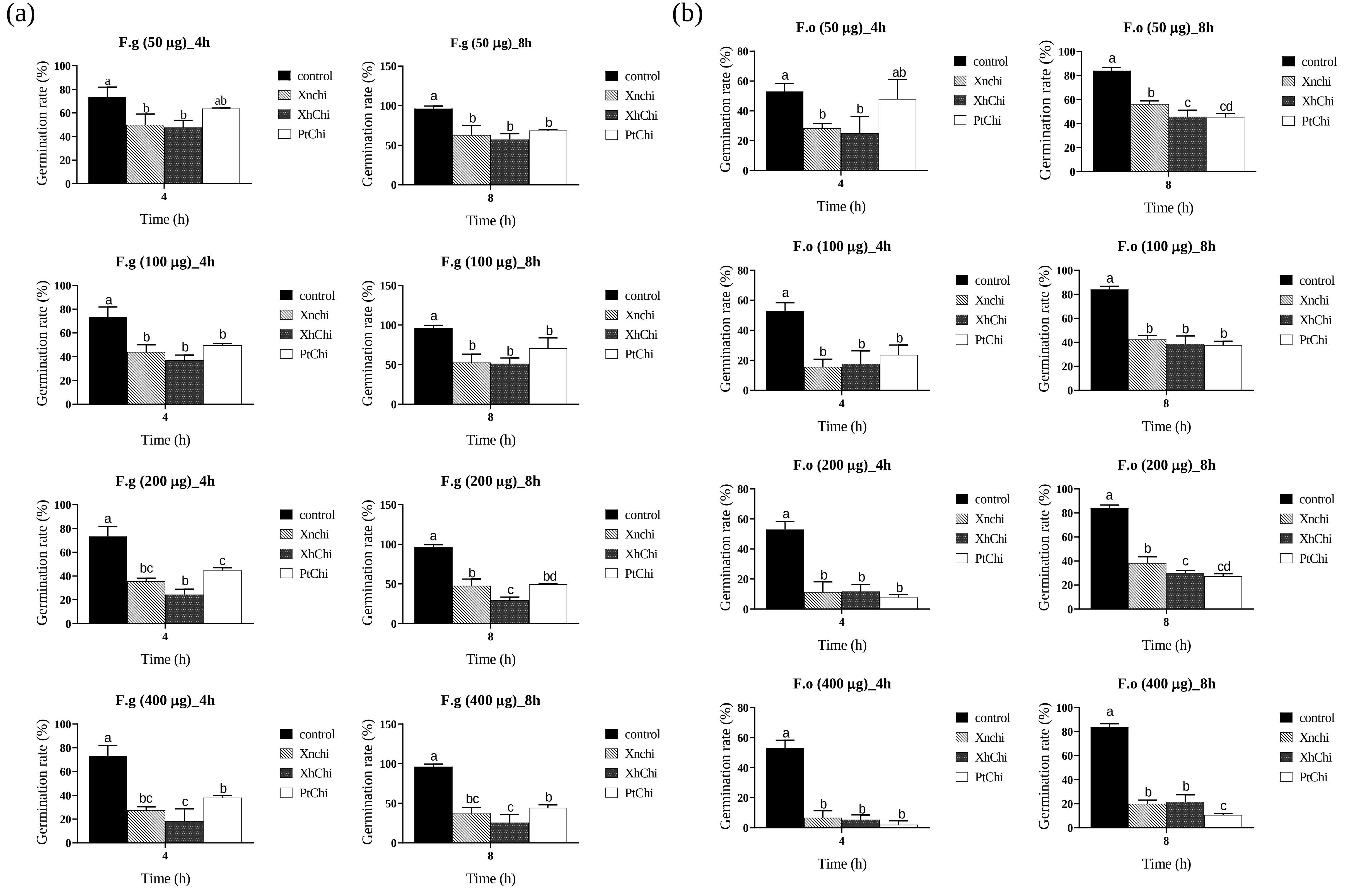

Figure S4. Effect of chitinase on conidial germination of *F.graminearum* (a) and *F.oxysporum* (b). Conidia were inoculated into PDB medium supplemented with or without chitinase in coculture system. Turkey's test was used as *post hoc* method to identify concentration and time of high antifugal activity that showed significant differences.

Table S1. Primer used in the study

**Primer**	Sequences (5' → 3')
X.nem chitinase-FW (*Sac I*)	GAGCTCATGTCTCAAAATGTTTATCGATACCC [1]
X.nem chitinase-RV (*Xba I*)	TCTAGACTACGATTTACGACGGGTTAC [1]
X.homi chitinase-FW (*Sac I*)	GAGCTCATGTCCAAGTTAACTCAAATAGATCCA [1]
X.homi chitinase-RV (*Sal I*)	GTCGACTTATTTGCTTGTTTTCCCTTTATAATAGAAAGG [1]
P.tem chitinase-FW (*Sac I*)	GAGCTCATGACTGATAACAGTAAATATCAATATACCTGC [1]
P.tem chitinase-RV (*Pst I*)	CTGCAGTTACTTTTTACGGGAATTAACATCAGG [1]

[1] The underlined part represents the enzyme cleavage sites.

Table S2. Amino acid sequences of chitinases of type strain used in the study

Strain name	Protein name	GeneBank access	Sequence
Xenorhabdus nematophia ATCC 19061	Glycosyl hydrolase family 18 protein	WP 010846090.1	MSQNVYRYPSIKAMSDASSEVGASLVAWQNQSGGQTWYVIYDSAVFKNIGWVERWHIPDRNISPDLPVY<br>ENAWQYVREATPEEIADHGNPNTPDVPPGEKTEVLQYDALTEETYQKVGYKPDGSGTPLSYSSARVAKSL<br>YNEYEVDPENTEPLPKVSAYITDWCQYDARLSPETQDNTALTSDDAPGRGFDLEKIPPTAYDRLIFSFMAV<br>NGDKGKLSERINEVVDGWNRQAEASSGQIAPITLGHIVPVDPYGDLGTTRNVGLDADQRRDASPKNFLQ<br>YYNQDAASGLLGGLRNLKARAKQAGHKLELAFSIGGWSMSGYFSVMAKDPEQRATFVSSIVDFFRRFP<br>MFTAVDIDWEYPGATGEEGNEFDPEHDGPNYVLLVKELREALNIAFGTRARKEITIACSAVVAKMEKSSFK<br>EIAPYLDNIFVMTYDFFGTGWAEYIGHHTNLYPPRYEYDGDNPPPPNPDRDMDYSADEAIRFLLSQGVQP<br>EKIHLGFANYGRSCLGADLTTRRYNRTGEPLGTMEKGAPEFFCLLNNQYDAEYEIARGKNQFELVTDTET<br>DADALFNADGGHWISLDTPRTVLHKGIYATKMKLGGIFSWSGDQDDGLLANAAHEGLGYLPVRGKEKID<br>MGP LYNKGRLIQLPKVTRRKS
Xenorhabdus hominickii ANU1	Glycosyl hydrolase family 18 protein	WP 010846090.1	MSKLTQIDPLSEESYRLDHFNPATETTKFSYTSGRVSLPVYNKYNVKNKPKVIGYYTDWSQYDSRLQGNQ<br>TPSARGRGIDLQHFLDNPFAYDKLIIGFCGILGDKGEKGSLISRAAPKFARYNNGEVTFTDEWGDCQSYVN<br>CTFPKWQDIQMPRDFNQAKAMGILGGLAKVQGAAKAQGHDLAMSFSVGGWTMSNGFYNMVRDKKLK<br>SSFISSLIDIFNRFPMFTEVDIDWEYPGASGNNNPHDDKDGEYYISLISDLSTEFKSAGRSDIKISIACSAVPA<br>VMEKSRIPELLNAGLYGINVMTYDFFGTPWANKLMHHTNLRRYPGSENSVEKAVEYLLSVGVPSQAINIG<br>YAGYSRNAKNVDITNYSPLSGSYHPGNGTTTGTFESGSTEWYDTIYNYLDLESRKDRNLFTLYTDEVADA<br>DYLYNSSSKLFLSIDTPRTVKAKAKYALEKNLGGVFTWTADQDRGLLVNAAREGLECPLLIKKIDMAPFY<br>YKGKTSK
Portorhabdus temperata J3	Glycosyl hydrolase family 18 protein	WP 023045972.1	MTDNSKYQYTCKKVMSDASENIGAPLTAWSNQSGGETYYVIFDGQVYKNTYWVERWHIPQRDNTPSYE<br>NAWSWVRAATAEEIAKHGNPKESTVKPIPGDVAILKPDALTEETYQKEGYKPDGSGTNLAYTSARVCHSL<br>YNQYETDRTRPKVSAYITDWCQYDARLPPASKDSDKNEDGPGRGFNLADIPATAYDRLVFSFLGIYGDKG<br>SKKDRINLSAEGWNKQLRPEDPPITFGHIVPVDPYGDLGTTRNVGLPKEDTRDAGPNTFLPFYNQQAASG<br>LLGGLRNLQQQAKLAGHRLELAFSIGGWSMSGYFSPMLKDDAQRKTFIDSIVDFFQRFPMFTAVDIDWEY<br>PGAAGEVGNEFDPENDGPNYAILIKKLRQALDCAFGTSARKQITIASSAVVGKLKKSNIKELIHNGLDNIFV<br>MTYDFFGNGWAKHIGHHTNLYSAPYASDDPNRDYDISSDEAIKYLIEVEGVPPGKIHLGFANYGRSCIGAD<br>LKTRQYNRNPANPAPALGTMENGAPEFFCLLNNQFDCEQQLAWAKNGFKLMTDTATDADFLYNSTGGHF<br>ISLDTPRTVFKKGIYATEHKLGGIFSWSGDQDCGLLANAAREGAGYIPIKGKEKIDMGPLYNRGELVELPD<br>VNSRKK

Table S3. Amino acid sequences of chitinases used in study

Strain name	Protein name	GeneBank access	Sequences
Xenorhabdus nematophia GJ1-2	XnChi	OR724704	MSQNVYRYPSIKAMSDASSEVGASLVAWQNQSGGQTWYVIYDSAVFKNIGWVERWHIPDRNISPDLPVYENAWQ<br>YVREATPEEIADHGNPNTPDVPPGEKTEVLQYDALTEETYQKVGYKPDGSGTPLSYSSARVAKSLYNEYEVDPEN<br>TEPLPKVSAYITDWCQYDARLSPETQDNTALTSDDAPGRGFDLEKIPPTAYDRLIFSFMAVNGDKGKLSERINEVV<br>DGWNRQAEASSGQIAPITLGHIVPVDPYGDLGTTRNVGLDADQRRDASPKNFLQYYNQDAASGLLGGLRNLKA<br>RAKQAGHKLELAFSIGGWSMSGYFSVMAKDPEQRATFVSSIVDFFRRFPMFTAVDIDWEYPGATGEEGNEFDPEH<br>DGPNYVLLVKELREALNIAFGTRARKEITIACSAVVAKMEKSSFKEIAPYLDNIFVMTYDFFGTGWAEYIGHHTNL<br>YPPRYEYDGDNPPPPNPDRDMDYSADEAIRFLLSQGVQPEKIHLGFANYGRSCLGADLTTRRYNRTGEPLGTMEK<br>GAPEFFCLLNNQYDAEYEIARGKNQFELVTDTETDADALFNADGGHWISLDTPRTVLHKGIYATKMKLGGIFSWS<br>GDQDDGLLANAAHEGLGYLPVRGKEKIDMGPLYNKGRLIQLPKVTRRKS
Xenorhabdus hominickii GJ11-1	XhChi	OR724706	MSKLTQIDPLSEESYRLDHFNPATETTKFSYTSGRVSLPVYNKYNVKNKPKVIGYYTDWSQYDSRLQGNQTPSAR<br>GRGIDLQHFLDNPFAYDKLIIGFCGILGDKGEKGSLISRAAPKFARYNNGEVTFTDEWGDCQSYVNCTFPKWQDIQ<br>MPRDFNQAKAMGILGGLAKVQGAAKAQGHDLAMSFSVGGWTMSNGFYNMVRDKKLKSSFISSLIDIFNRFPMF<br>TEVDIDWEYPGASGNNNPHDDKDGEYYISLIRDLSMEFKSAGRSDIKISIACSAVPAVMEKSRIPELLNAGLYGINV<br>MTYDFFGTPWANKLMHHTNLRRYPGSENSVEKAVEYLLSVGVPSQAINIGYAGYSRNAKNVDITNYSPLSGSYHP<br>GNGTTTGTFESGSTEWYDTIYNYLDLESRKDRNLFTLYTDEVADADYLYNSSSKLFLSIDTPRTVKAKAKYALEK<br>NLGGVFTWTADQDRGLLVNAAREGLECPLLIKKIDMAPFYYKGKTSK
Portorhabdus temperata GJ1-2	PtChi	OR724705	MTDNSKYQYTCKKVMSDASENIGAPLTAWSNQSGGETYYVIFDGQVYKNTYWVERWHIPQRDNTPSYENAWS<br>WVRAATAEEIAKHGNPKESTVKPIPGDVAILKPDALTEETYQKEGYKPDGSGTNLAYTSARVCHSLYNQYETDRT<br>RPKVSAYITDWCQYDARLPPASKDSDKNEDGPGRGFNLADIPATAYDRLVFSFLGIYGDKGSKKDRINLSAEGWN<br>KQLRPEDPPITFGHIVPVDPYGDLGTTRNVGLPKEDTRDAGPNTFLPFYNQQAASGLLGGLRNLQQQAKLAGHRL<br>ELAFSIGGWSMSGYFSPMLKDDAQRKTFIDSIVDFFQRFPMFTAVDIDWEYPGAAGEVGNEFDPENDGPNYAILIK<br>KLRQALDCAFGTSARKQITIASSAVVGKLKKSNIKELIHNGLDNIFVMTYDFFGNGWAKHIGHHTNLYSAPYASDD<br>PNRDYDISSDEAIKYLIEVEGVPPGKIHLGFANYGRSCIGADLKTRQYNRNPANPAPALGTMENGAPEFFCLLNNQ<br>FDCEQQLAWAKNGFKLMTDTATDADFLYNSTGGHFISLDTPRTVFKKGIYATEHKLGGIFSWSGDQDCGLLANAA<br>REGAGYIPIKGKEKIDMGPLYNRGELVELPDVNSRKK

Table S4. Amino acid sequences of chitinases used in multiple sequence alignment analysis.

Strain name	Protein name	GeneBank access	Sequence
Yersinia entomophaga MH96	Glycosyl hydrolase family 18 protein	ANI28952.1	MESEMEKEEKSNLIYDKDPGYVWDNKNECEGAAEETYQELNYEPSISADKLTWTPTRLAKTVFNTYEDD<br>DDFNVLCYFTDWSQYDPRIINKEIRDTGGRSADILRLNTPDGRPFKRLIYSFGGLIGDKKYSADGNASIAVR<br>LGVATDPDDAIANHKGKTIPVDPDGAVLASINCGFTKWEAGDANERYNQEKAKGLLGGFRLLHEADKEL<br>EFSLSIGGWSMSGLFSEIAKDEILRTNFVEGIKDFFQRFPMFSHLDIDWEYPGSIGAGNPNSPDDGANFAILI<br>QQITDAKISNLKGISIASSADPAKIDAANIPALMDAGVTGINLMTYDFFTLGDGKLSHHTNIYRDPSDVYSK<br>YSIDDAVTHLIDEKKVDPKAIFIGYAGYTRNAKNATITTSIPSEEALKGTYTDANQTLGSFEYSVLEWTDIIC<br>HYMDFEKGEGRNGYKLVHDKVAKADYLYSEATKVFISLDTPRSVRDKGRYVKDKGLGGLFIWSGDQDN<br>GILTNAAHEGLKRRIKNKVIDMTPFYLDSDEELPTYTEPAEPQCEACNIK
Pseudomonas chloriraphis B25	Chitinase	WP_124321822.1	MYDEKKYQYTSKKAMSDASEGVGNSLAAWNNQSGGETFYVIFDSGVYTNSYWVERWHVPDRNLESPS<br>HENAWTYVRPATPQEMAEHGNPTEGIVEPSPDKTQILQPDDLTEKTYVDVNYFPDGSGSNLSYTAARVCK<br>SMYNEYEQDRTRDRPHLKVSAYVTDWCQYDARLPPESRALQPAEDVKPGRGFDLENIPPTAYDRLVFSFL<br>AIYGDKGKLGKEIEAVADGWNSQVEADEAITHGHIVPVDPYGDLGTTRNVAVPPDDRREVGPHNFLQYY<br>NQEAASGLLGGLRNLQERAKKVGHKLELAFSIGGWSMSGYFSPMVKDPQQRRAFVESVVDFFRRFPMF<br>TAVDIDWEYPGTSGEEGNEWDEEHDGPNYAILVGELRKALDGAFGTRARKEITIASSAVVGKLKKSNIRGL<br>IENGLDNIFVMTYDFFGTGWADWIGHHTNLYAPPYAENNPDRLYDLSADEAIQYLINDEGVPPGKIHLGFA<br>NYGRSCIGANLSDRTYTKPGMALGTMENGAPEFFCLLNNQFDCEHQLALGKNGFKLMTDTAADADILFN<br>PVGNHYISLDTPRTVMQKGIYADKEKLGGIFSWSGDQDDGLLANAAREGTGYIPKKGREVIDMGPLYNR<br>GELFELPATDQGKIKTAARKRKISKD
Cronobacter sakazakii wls2261	Glycosyl hydrolase family 18 protein	WP_241700376.1	MATSKLIQGDSLTETSNAADGFNPASETEKYSYTSARVAKPVYNKYKAANKPKVFGYYTDWSQYDGRLQ<br>GDDSPANRGRGYDLAKVSPTAYDKIVFGFLGIVGDKGEKQYTIENAARQTNKNTNEPTFLDPWGDFQSY<br>ENCGHTTSGWDIDPATVTQATTKGLLGGLRDLQQKAKQQGHTLVLSMSIGGWTMSNGFHEMSKTDSSR<br>KTFAAGVVKLFKQFPMFSEVDIDWEYPNAEGNGNPHGPEDGANYALLIAEMKKQLSAAGRSDVKISIASS<br>AVVSTLEYSNVKALLDAGLYAINVMTYDFFGTPWADTLTHHTNLTPLTAGGWSIEAVVDYLIAEGFPSDRI<br>NIGYAGYTRNARNAVIESFSPLKGSYSPGNGTTTGSFESGSTEWYDTIYNYLDLENQKGRNGFNVYTDKV<br>ANADYLYNPQSKLFMSLDTPRSVKAKGQYAASRNLGGLFTWTIDQDNGVLVNAAREGLGYEIDTQTIDM<br>KPFYFEGINVAEPDNSGGDSEPQPTVNHAPVAAIQLR

Table S5. Amino acid sequences of chitinases used in phylogenetic analysis.

Photorhabdus khanii HGB1456	Glycosyl hydrolase family 18 protein	WP 152962976.1	MTDNSKYQYTCKKVMSDASENIGAPLTAWSNQSGGETYYVIFDGQVYKNTYWVERWHIPQRDNTPSYE<br>NAWSWVRAATAEEIAKHGNPKEGTVKPIPGDVAILKPDALTEETYQKEGYKPDGSGTNLAYTSARVCHSL<br>YNQYETDRTRPKVSAYITDWCQYDARLPPASKDSDKNEDGPGRGFNLADIPATAYDRLVFSFLGIYGDKG<br>SKKDRINLSAEGWNKQLRPEDPPITFGHIVPVDPYGDLGTTRNVGLPKEDTRDAGPNTFLPFYNQQAASG<br>LLGGLRNLQQQAKLAGHRLELAFSIGGWSMSGYFSPMLKDDAQRKTFIDSIVDFFQRFPMFTAVDIDWEY<br>PGAAGEVGNEFDPENDGPNYAILIKKLRQALDCAFGTSARKQITIASSAVVGKLKKSNIKELIHNGLDNIFV<br>MTYDFFGNGWAKHIGHHTNLYSAPYASDDPNRDYDISSDEAIKYLIEVEGVPPGKIHLGFANYGRSCIGAD<br>LKTRQYNRNPDNPAPALGTMENGAPEFFCLLNNQFDCEQQLAWAKNGFKLMTDTATDADFLYNSTGGHF<br>ISLDTPRTVFKKGIYATEHKLGGIFSWSGDQDCGLLANAAREGAGYIPIKGKEKIDMGPLYNRGELVELP D<br>VNSRKK
Pseudomonas chlororaphis B25	Glycosyl hydrolase family 18 protein	WP 124321822.1	MYDEKKYQYTSKKAMSDASEGVGNSLAAWNNQSGGETFYVIFDSGVYTNSYWVERWHVPDRNLESPS<br>HENAWTYVRPATPQEMAEHGNPTEGIVEPSPDKTQILQPDDLTEKTYVDVNYFPDGSGSNLSYTAARVCK<br>SMYNEYEQDRTRDRPHLKVSAYVTDWCQYDARLPPESRALQPAEDVKPGRGFDLENIPPTAYDRLVFSFL<br>AIYGDKGKLGKEIEAVADGWNSQVEADEAITHGHIVPVDPYGDLGTTRNVAVPPDDRREVGPHNFLQYY<br>NQEAASGLLGGLRNLQERAKKVGHKLELAFSIGGWSMSGYFSPMVKDPQQRRAFVESVVDFFRRFPMF<br>TAVDIDWEYPGTSGEEGNEWDEEHDGPNYAILVGELRKALDGAFGTRARKEITIASSAVVGKLKKSNIRGL<br>IENGLDNIFVMTYDFFGTGWADWIGHHTNLYAPPYAENNPDRLYDLSADEAIQYLINDEGVPPGKIHLGFA<br>NYGRSCIGANLSDRTYTKPGMALGTMENGAPEFFCLLNNQFDCEHQLALGKNGFKLMTDTAADADILFN<br>PVGNHYISLDTPRTVMQKGIYADKEKLGGIFSWSGDQDDGLLANAAREGTGYIPKKGREVIDMGPLYNR<br>GELFEL PATDQGKIKTAARKRKISKD
Xenorhabdus nematophila ATCC19061	Glycosyl hydrolase family 18 protein	WP 010846090.1	MSQNVYRYPSIKAMSDASSEVGASLVAWQNQSGGQTWYVIYDSAVFKNIGWVERWHIPDRNISPDLPVY<br>ENAWQYVREATPEEIADHGNPNTPDVPPGEKTEVLQYDALTEETYQKVGYKPDGSGTPLSYSSARVAKSL<br>YNEYEVDPENTEPLPKVSAYITDWCQYDARLSPETQDNTALTSDDAPGRGFDLEKIPPTAYDRLIFSFMAV<br>NGDKGKLSERINEVVDGWNRQAEASSGQIAPITLGHIVPVDPYGDLGTTRNVGLDADQRRDASPKNFLQ<br>YYNQDAASGLLGGLRNLKARAKQAGHKLELAFSIGGWSMSGYFSVMAKDPEQRATFVSSIVDFFRRFP<br>MFTAVDIDWEYPGATGEEGNEFDPEHDGPNYVLLVKELREALNIAFGTRARKEITIACSAVVAKMEKSSFK<br>EIAPYLDNIFVMTYDFFGTGWAEYIGHHTNLYPPRYEYDGDNPPPPNPDRDMDYSADEAIRFLLSQGVQP<br>EKIHLGFANYGRSCLGADLTTRRYNRTGEPLGTMEKGAPEFFCLLNNQYDAEYEIARGKNQFELVTDTET<br>DADALFNADGGHWISLDTPRTVLHKGIYATKMKLGGIFSWSGDQDDGLLANAAHEGLGYLPVRGKEKID<br>MGP LYNKGRLIQLPKVTRRKS

Photorhabdus laumondii subsp. *laumondii* TTO1	Chitinase	AXG43067.1	MVNKYTYTSSKSMSDISDIVGEPLTAWNNQAGGQVYNVIFNSGVYTNTYWVERWHIPDPTAKEGTPHNA<br>WKYIRPATEDEIKQHGNPTDGRIDPTEDIPSPVLQPDAITEQTYQRPDVNFKPDGSAGNLSYTASRVCRPM<br>YNEYESDKSRPKLSAYITDWCQYDARLDGKNEDADQRGRGFDLSTINVAAYDKLIFSFLGIYGDTGVKGD<br>KIKEVADGWNSQSEIKITEGHIVPLDPYGDLGTARNVGLPKESANTNINAGTFLPFYQQKKASGLLGGLRE<br>LQKTARLAGHKLELAFSIGGWSMSGYFSVMAADETKRGVFVDSIVDFFARFPMFSCVDIDWEYPGSPGE<br>DGNVYDPKDGEHYALLIKELRESLDSRFGREDRKEISIACSGVKTKLATSNIAELVKNGLDNIYLMSYDYF<br>GTGWAPYIGHHTNLYSPKDPDPSGASDLSAEVAINYLHQELGIPLEKIHLGYANYGRAGKGANLETREYN<br>KTGEALGTMEKGSPEFFDIVNNYLDSEHTLATGKNGFVLMTDTNADADFLFSETEGHFISLDTPRTVKQK<br>AEYVATNKLGGIFSWSGDQDCGLLANAAREGMGYIAKSNDETIDMGPLYNPGKPYYLKSIG
Photorhabdus akhurstii DSM 15138	Glycosyl hydrolase family 18 protein	WP 214039151.1	MVNKYTYTSSKSMSDISDIIGEPLTAWKNQAGGQVYNVIFNGGVYTNTYWVERWHIPDPTAKEGTPHNA<br>WKYIRQATEEEIKEHGNPTDGRVDPTEDIPSPVLQPDDITEKTYQRPDVNFKPDGSAGNLSYTATRVCRPM<br>FNEYESDKSRPKLSAYITDWCQYDARLDGKDDEADDRGRGFDLSTINVTAYDKLIFSFLGIYGDTGVKGK<br>TIKEVADGWNSQSEIKITEGHIVPLDPYGDLGTARNVGLPKESANTDINAGTFLPFYQQKKASGLLGGLRE<br>LQKTARLAGHKLELAFSIGGWSMSGYFSVMAADETKRGVFVDSIVDFFERFPMFSCVDIDWEYPGSPGE<br>DGNVYDPKDGEYYALLIKELRESLDSRFGREDRKEISIACSGVKTKLETSNIAELVKNGLDNIYLMSYDYF<br>GTGWAPYIGHHTNLYSPKDPDPSGASDLSAEVAINYLHQDLGIPLEKIHLGYANYGRAGKGAKLETREYN<br>KTGDALGTMEKGSPEFFDIVNNYLDSEHTLATGKNGFVLMTDTNADADFLFSETAGHFISLDTPRTVKQK<br>AEYVAKNKLGGIFSWSGDQDCGLLANAAREGMGYVAKSNDETIDMGPLYNPGKPYYLKSIG
Yersinia entomophaga MH96	Chitinase	ANI28955.1	MVNKYTYTSSKAMSDISDVIGEPLAAWDSQVGGRVFNVIFDGKVYTNTYWVERWQVPGIGSSDGNPHN<br>AWKFVRAATADEINKIGNPTTADVKPTENIPSPILVEDKYTEETYSRPDVNFKEDGSQGNLSYTATRVCAP<br>MYNHYVGDKTKPKLSAYITDWCQYDARLDGGGSKEEERGRGFDLATLMQNPATYDRLIFSFLGICGDIG<br>NKSKKVQEVWDGWNAQAPSLGLPQIGKGHIVPLDPYGDLGTARNVGLPPESADTSIESGTFLPYYQQNR<br>AAGLLGGLRELQKKAHAMGHKLDLAFSIGGWSLSSYFSALAENPDERRVFVASVVDFFVRFPMFSCVDI<br>DWEYPGGGGDEGNISSDKDGENYVLLIKELRSALDSRFGYSNRKEISIACSGVKAKLKKSNIDQLVANGL<br>DNIYLMSYDFFGTIWADYIGHHTNLYSPKDPGEQELFDLSAEAAIDYLHNELGIPMEKIHLGYANYGRSAV<br>GGDLTTRQYTKNGPALGTMENGAPEFFDIVKNYMDAEHSLSMGKNGFVLMTDTNADADFLFSEAKGHF<br>ISLDTPRTVKQKGEYAAKNKLGGVFSWSGDQDCGLLANAAREGLGYVADSNQETIDMGPLYNPGKEIYL<br>KSISEIKSK

Yersinia nurmii CIP110231T	Insecticidal toxin complex chitinase Chi2	WP 049597577.1	MVNKYTYTSSKAMSDISDVIGEPLAAWNSQVGGRVFNVIFDSKVYTNTYWVERWQVPGIGSSDGNPHN<br>AWKFVRAATVDEINKIGNPTTADVKPTENIPSPILVEDKYTEETYSRPDVNYKEDGSQGNLSYTATRVCAP<br>MYNHYVGDKTKPKLSAYITDWCQYDARLDGGGSKEEERGRGFDLATLMQNPATYDRLIFSFLGICGDIG<br>DKSKKVQEVWDGWNAQASSLGLPQISKGHIVPLDPYGDLGTARNVGLPLESADTSIESGTFLPYYQQNR<br>AAGLLGGLRELQKKAHAMGHKLDLAFSIGGWSLSSYFSALAENPDERRVFVASVVDFFERFPMFSCVDID<br>WEYPGGGGEAGNISSDKDGENYVLLIKELRSALDSRFGYSNRKEISIACSGVKAKLKKSNIDQLVANGLD<br>NIY<br>LMSYDFFGTIWADYIGHHTNLYSPKDPGEQELFDLSAEAAIDYLHNELAIPMEKIHLGYANYGRSAVGGD<br>LTTRQYTKDGPALGTMENGAPEFFDIVRNYMDVEHSLSTGKNGFVLMTDTNADADFLFSEAKGHFISLD<br>TPRTVKQKGEYAAKNKLGGVFSWSGDQDCGLLANAAREGLGYVTQSNQETIDMGPLYNPGKEIYLKSIS<br>EIKSK
Xenorhabdus lircayensis VLS	Glycosyl hydrolase family 18 protein	WP 198689572.1	MSKITQIDPLSEESYRLDNFKPATETTKYSYTSGRVALPVYNKYDVKNRPKVIGYYTDWSQYDGRLQGNQ<br>TPSARGRGIDLQHLLDNPFAYDKLIIGFCGILGDKGEKASLISRAAPKFDRHKNGEVTFTDEWGDCQSYVN<br>CTYPGWQDIHMPRDFNQANSMGILGGLAKVQGAARAQGHDLAMSFSVGGWTMSNGFFHMVRDNQLK<br>SNFISSLIDIFRRFPMFTEVDIDWEYPGAPGNQNPHDSDDGKYYISLIRDLSSAFKNEGRSDIKISIACSAVPD<br>IMEKSSIPELLNAGLYGVNVMTYDFFGTPWADKLRHHTNLRRYPGSENSVEKAVEYLLSVGVPSQAINIG<br>YAGYSRNAKNTNIDSYSPLSGSYNHDKGTTTTGTFESGSTEWYDTIYNYIDLESRKDRNNFTLYTDEVAD<br>ADYLYNQDSKLFLSIDTPRTVKAKAKYALEKNLGGVFTWTADQDRGLLVNAAREGLECPLLIKKIDMEPF<br>YYKGKTSK
Photorhabdus laumondii subsp. *laumondii* TTO1	Chitinase	AXG42878.1	MSKIIQTDSFSEQSYQIDNFNPSTETINFSYTSGRIALPVYNKYDTRNKPKVFGYYTDWSQYDGRLQGNQ<br>DPIRRGRGIDLQHLLDNPFAYDKLVIGFCGILGDEGEKKQQIKEQAPLFARTNNGEVTFTDAWGDCQSYQN<br>CTYSGWQDIQMPRDFDQSKSMGVLGGLAKVQQAAKAQGHDLIMSFSVGGWTMSNGFYAMVRDETLKS<br>YFCSSLVDIFQRFPMFTEIDIDWEYPGVAGNEGNMYASDDGDYYVSLIKDLTAAFKEAGRSDIKISIACSAV<br>PEKMEKSKIPELLQVGLYGVNVMTYDFFGTPWAERLLHHTNLRNYPSGENSVEKAVEYLLSLGVPASAIN<br>IGYTGYSRNGRNTVIDSYSPLSGSYDPGYVPKKEPKITTTGSFESGTTEWYDTIYNYLDLEEKRGRNNFDL<br>YTDEIADADYLYNKDSQLFISLDTPRSVKSKAQYALEKNLGGIFTWTADQDRGLLVNAAREGLGCELITE<br>KIDMTPFYFKGVNVEPVDPIHPDTGSDYPVWDANRAYKAGDRVSWNNQNWEAKWWNRGTEPDGAGT<br>SDAYPWVKIA

Yersinia proxima IP37424	Glycosyl hydrolase family 18 protein	WP 227731375.1	MSLLIQYDEATEKSYDLDGFSAKTETNNYSYTSARVAKSVYNKYNVKGRAKVIGYYTDWSQYDGRLDN<br>NQQKSLRGRGVDLALVDPLAYDKLIIGFCGIVGDQGDKHQLIAQQSPLFGRFYPGEASFIDAWGDCQSYR<br>NCGFPAYQDIPMPQGFNQLNAMGVLGGLRDLQRKALEQGHELALSFSIGGWTMSNAFHDMVRDIHLRQ<br>VFCRSVVDIFTRFPMFSEVDIDWEYPGSAGNNNPFDENDGKCYALLIKELKGMLVAANRPDVKISIACSAI<br>PAIMAKSNIPELISAGLYGLNVMTYDFFGTPWSELVTHHTNLHSTPETSYSVESAVDYLLAQGVPAEAINIG<br>YAGYSRNAKMAKLSTLSPLSGNYNPGDGNTTGTFDSGSTEWYDIINNYLDLENRKGRNGFNLYTDEVAD<br>ADYLYNPESSLFISIDTPRTVKAKAEYVKNKNLGGIFTWTADQDQGLLVNAAREGLECPVVKQNIDMSPF<br>YFKGINVTPSPIEAIRTVITGPTISKIGDKVTLSGENSSSATPGSLQFTWITPDSVNPDAKDKSVISFIVPSVNS<br>SMEKLQFMLSVTDGVDHATVSHSISVVSPPDYPEWQKEATYIASDRVSWKGDNWEAKWWTYAAEPGTA<br>DPTDAYPWKKLI
Cronobacter sakazakii wls2261	Glycosyl hydrolase family 18 protein	WP 241700376.1	MATSKLIQGDSLTETSNAADGFNPASETEKYSYTSARVAKPVYNKYKAANKPKVFGYYTDWSQYDGRLQ<br>GDDSPANRGRGYDLAKVSPTAYDKIVFGFLGIVGDKGEKQYTIENAARQTNKNTNEPTFLDPWGDFQSY<br>ENCGHTTSGWDIDPATVTQATTKGLLGGLRDLQQKAKQQGHTLVLSMSIGGWTMSNGFHEMSKTDSSR<br>KTFAAGVVKLFKQFPMFSEVDIDWEYPNAEGNGNPHGPEDGANYALLIAEMKKQLSAAGRSDVKISIASS<br>AVVSTLEYSNVKALLDAGLYAINVMTYDFFGTPWADTLTHHTNLTPLTAGGWSIEAVVDYLIAEGFPSDRI<br>NIGYAGYTRNARNAVIESFSPLKGSYSPGNGTTTGSFESGSTEWYDTIYNYLDLENQKGRNGFNVYTDKV<br>ANADYLYNPQSKLFMSLDTPRSVKAKGQYAASRNLGGLFTWTIDQDNGVLVNAAREGLGYEIDTQTIDM<br>KPFYFEGINVAEPDNSGGDSEPQPTVNHAPVAAIQLR
Photorhabdus laumondii subsp. *laumondii* DJC	Chitinase	AXG43070.1	MATSKLIQGDSLTETSNAADGFNPASETEKYSYTSARVAKPVYNKYKAANKPKVFGYYTDWSQYDGRLQ<br>GDDSPANRGRGYDLAKVSPTAYDKIVFGFLGIVGDKGEKQYTIENAARQTNKNTNEPTFLDPWGDFQSY<br>ENCGHTTSGWDIDPATVTQATTKGLLGGLRDLQQKAKQQGHTLVLSMSIGGWTMSNGFHEMSKTDSSR<br>KTFAAGVVKLFKQFPMFSEVDIDWEYPNAEGNGNPHGPEDGANYALLIAEMKKQLSAAGRSDVKISIASS<br>AVVSTLEYSNVKALLDAGLYAINVMTYDFFGTPWADTLTHHTNLTPLTAGGWSIEAVVDYLIAEGFPSDRI<br>NIGYAGYTRNARNAVIESFSPLKGSYSPGNGTTTGSFESGSTEWYDTIYNYLDLENQKGRNGFNVYTDKV<br>ANADYLYNPQSKLFMSLDTPRSVKAKGQYAASRNLGGLFTWTIDQDNGVLVNAAREGLGYEIDTQTIDM<br>KPFYFEGINVAEPDNSGGDSEPQPTVNHAPVAAIQLR

Yersinia entomophaga MH96	Chitinase	ANI28952.1	MESEMEKEEKSNLIYDKDPGYVWDNKNECEGAAEETYQELNYEPSISADKLTWTPTRLAKTVFNTYEDD<br>DDFNVLCYFTDWSQYDPRIINKEIRDTGGRSADILRLNTPDGRPFKRLIYSFGGLIGDKKYSADGNASIAVR<br>LGVATDPDDAIANHKGKTIPVDPDGAVLASINCGFTKWEAGDANERYNQEKAKGLLGGFRLLHEADKEL<br>EFSLSIGGWSMSGLFSEIAKDEILRTNFVEGIKDFFQRFPMFSHLDIDWEYPGSIGAGNPNSPDDGANFAILI<br>QQITDAKISNLKGISIASSADPAKIDAANIPALMDAGVTGINLMTYDFFTLGDGKLSHHTNIYRDPSDVYSK<br>YSIDDAVTHLIDEKKVDPKAIFIGYAGYTRNAKNATITTSIPSEEALKGTYTDANQTLGSFEYSVLEWTDIIC<br>HYMDFEKGEGRNGYKLVHDKVAKADYLYSEATKVFISLDTPRSVRDKGRYVKDKGLGGLFIWSGDQDN<br>GILTNAAHEGLKRRIKNKVIDMTPFYLDSDEELPTYTEPAEPQCEACNIK
Xenorhabdus nematophila HB310	Chitinase 60	AGK44778	MPSSKNSNLNDNPFVIIPDPGTETKPGDIWGAGATEKTYEVNEFDPSTADSDLSYTPGRIAKNVFNHYESV<br>SGFEVFGYLSDWGIYDSRYGNAPGDTDVDYGEGGRGTDIMRLLDEGSPLPYFDRIIVGFAGIIGDEGLKEA<br>TINQAAIDFKIASDEDDLPNHKGEATFTDYWGDTGAYLNCGFPGWKETDFTPENAQGVLGALVKLHKKY<br>PDMPIGLSLGGWSMSQAFHFIAQEPELRQRLAQSLKKIFDLFPMFTDLDLDWEYPNYKGEEHNSYDEEDP<br>ENFAELIKEIRKELPDITISIATIAVPAGLEAANIPLLLEAGVDKLNVMTYDFFGTPWAETLGHHTALKLNPD<br>KEETQNSVDKAVNYLLDELHVEPKKINIGYAGYTRNAQQASIPSISPIYGRYTPRGDIALGSFESGSTEWPD<br>LLRNYLDSNMDGINNFTVYTDEVAKAEFLYNQESRLFMSLDTPYSVKEKAQYVKEKGLGGMFIWMIDH<br>DNGLLTNAAREGLGAATVGTPRVDMAPLCLSAAEKAKNRTK
Pseudomonas fluorescens MP-13	Chitinase	AIU96334.1	MSKFDFTLLKSAVSDAASLMPSIAGKKILMGIWHNWPAGPSDGYQRGQFANIALHDVPKDYNVVAVAFM<br>KGNGIPTFNPYNLSDAEFRRQVGVLNSQGRAVLISLGGADAHIELHKGNEQPLANEIIRLVSTYGFDGLDI<br>DLEQSAIDFADNKTVLPAALKLVKDHYAGEGKHFIIIMAPEFPYLTTAGKYVGYLQALEGYYDFIAPQYYN<br>QRGDGIWVQEANNGNGAWIAQNNDAMKEDFLYYLTESLVSGTRGFTRIPADKFVIGLPDNVDAAATGYV<br>INPAAVVNAFKRLDAKGLSIKGLMTWSVNWDNAVNKDHVPYNWEFSRRYGPLINGKRLSWHEEALAAT<br>EVANTL

Pseudomonas protegens CHA0	Glycosyl hydrolase family 18 protein	WP 015634913.1	MDKIDFALIKSQAADAASLMPSIAGKKVLMGFWHNWAAGHSDGYQQGQFANLDLVDVPKEYNVVAVAFMKGNGIPTFKPYNLSDAEFRRQVGVLNSQGRAVLISLGGADAHIELHKGNEQPLANEIIRLVETYGFDGLDIDLEQSAIDFADNKSVLPAALKLVKDHYAGQGKHFIISMAPEFPYLTSNGKYVAYLQALEGYYDFVAPQFYNQGGDGLWVQEANGGQGAWIAQNNDAMKEDFLYYLSESLASGTRGFTRIAADKLVIGLPSNVDAAATGYVIDPSAVSNAFKRLQGAGHAIKGLMTWSVNWDAGISRQGVPYNWEFRHRYAPLIHGDGGEPERPGAPGNLMVLGTSRSSVNLSWGVSGSVRPVEFYTLYRDGNAVARTPSPGFEDQGLSADTQYSYFVTATDTQGQESLPSRSVSARTAGGAVDPSFPEWRTSQHYLREDGVTYEGNRYLCLQEHTSNPGWTPSVAFTLWSKVLQERHG
Serratia marcescens GEI	Chitinase B	ACX42072.1	MSTRKAVIGYYFIPTNQINNYTESDTSIVPFPVSNITPAKAKQLTHINFSFLDINSNLECAWDPATNDAKARDVVKRLTALKAHNPNLRIMFSIGGWYYSNDLGVSHANYVNAVKTPASRTKFAQSCVRIMKDYGFDGVDIDWEYPQAAEVDGFIAALQEIRTLLNQQTVADGRQALPYQLTIAGAGGAFFLSRYYSKLAQIVAPLDYINLMTYDLAGPWEKITNHQAALFGDAAGPTFYNALREANLGWSWEELTRAFPSPFSLTVDAAVQQHLMMEGVPSAKIVMGVPFYGRAFKGVSGGNGGQYSSHSTPGEDPYPSTDYWLVGCEECVRDKDPRIASYRQLEQMLQGNYGYQRLWNDKTKTPYLYHAQNGLFVTYDDAESFKYKAKYIKQQQLGGVMFWHLGQDNRNGDLLAALDRYFNAADYDDSQLDMGTGLRYTGVGPGNLPIMTAPAYVPGTIYAQGALVSYQGYVWQTKWGYITSAPGSDSAWLKVGRVA
Serratia proteamaculans 336x	ChiB	AGF70636.1	MSERKAVIGYYFIPTNQINNYTESDTSIVPFPVSNITPAKAKQLTHINFSFLDINSNLECAWDPATNDAKARDVVSRLTALKAHNPSLRIMFSIGGWYYSNDLGVSHANYVNAVKTPAARTKFAQSCVRIMKDYGFDGVDIDWEYPQSSEVDGFVAALQEIRTLLNQQTLTDGRQALPYQLTIAGAGGAFFLSRYYSKLPQIVASLDYINLMTYDLAGPWEKITNHQAGLFGDSAGPTFYNALREANLGWSWEELTRAFPSPFSLTVDAAVQQHLMLEGVPSNKIVMGVPFYGRAFKGVSSSNGGQYSSHSTPGEDPFPGTDYWLVGCEECVRDKDPRIASYRQLEQMLLGNYGYQRLWNDKTKTPYLYHAANGLFVTYDDVESFKYKAKYIKQQQLGGVMFWHLGQDNRNGDLLASLDRYFNAADYDDSQLDMGTGLRYTGVGPGNLPIMSAPAYVAGTTYNQGALVSYLGYVWQTKWGYITSVPGSDSAWLKVGRVA

Bacillus pumilus<br>MCB-7	Chitinase A	ALX35002.1	MNKVLVNKSKKFLVLSFISAMILSLSLFNGEVAKASSDKSYKIIGYYPSWGAYGRDYQVWDMDASKVSHI<br>NYAFADICWEGRHGNPDPTGPNPQTWSCQDENGVIDAPYGSIVMGDPWIDAQKSNAGDAWDEPIRGNSK<br>QLLKLKKNHPHLKTFISGGGWSWSNRFSDVAADPAARENFAASAVNLLRKYGFDGVDLDWEYPVSGGL<br>PGNSTRPEDKRNYTLLLQDVPKKLDAAEAKDGKKYLLTIASGASPEYVSNTELDKIAETVDWINSMTYDF<br>NGGWQSINAHNAPLFYDPKAKEAGVPNAETFNIESTVKRYKEAGVKADKLVLGTPFYGRGWSNCEPGD<br>NGEYQKCGPGKEGTWEKGVFDISDLEKNYINKNGYKRYWNDRAKVPFLYYAENGNFITYDDEESYGYK<br>TDLIQSNGLSGAMFWDFSGDSNQTLLNKLAADLGFAPGGRNPEPPSSAPENVRVTEKTAASVSLAWDAPS<br>DGANIAEYVLSYKYGSISVKETSAKIGQLKPNTTCSFTVSAKDADGKLHTGPTIEAATNSDQTCGYNEWK<br>DTAVYSVGDRVVFNGKVYEAKWWTKGEQPDQSGESGVWKLIGDCK
Bacillus thuringiensis	Chitinase	AAL17867.1	MAMRSQKFTLLLLSLLLFLPLFLTNFITPNLALADSPKQSQKIVGYFPSWGVYGRNYQVADIDASKLTHLN<br>YAFADICWNGKHGNPSTHPDNPNKQTWNCKESGVPLQNKEVPNGTLVLGEPWADVTKSYPVSGTTWED<br>CDKYARCGNFGELKRLKAKYPHLKTIISVGGWTWSNRFSDMAADEKTRKVFAESTVAFLRAYGFDGVDL<br>DWEYPGVETIPGGSYRPEDKQNFTLLLQDVRNALNKAGAEDGKQYLLTIASGASQRYADHTELKKISQIL<br>DWINIMTYDFHGGWEATSNHNAALYKDPNDPAANTNFYVDGAINVYTNEGVPVDKLVLGVPFYGRGW<br>KSCGKENNGQYQPCKPGSDGKLASKGTWDDYSTGDTGVYDYGDLAANYVNKNGFVRYWNDTAKVPY<br>LYNATTGTFISYDDNESMKYKTDYIKTKGLSGAMFWELSGDCRTSPKYSCSGPKLLDTLVKELLGGPINQ<br>KDTEPPTNVKNIVVTNKNSNSVQLNWTASTDNVGVTEYEITAGEEKWSTTTNSITIKNLKPNTEYTFSIIAK<br>DAAGNKSQPTALTVKTDEANTTPPDGNGTATFSVTSNWGSGYNFSIIIKNNGTTPIKNWKLEFDYSGNLTQ<br>VWDSKISSKTNNHYVITNAGWNGEIPSGGSITIGGAGTGNPAELLNAVISEN
Bacillus cereus<br>VD133	Glycosyl hydrolase<br>family 18 protein	WP 016111320.1	MRSQKFTLLLLSLLLFLPLFLTNFITPNLALADSPKQSQKIVGYFPSWGVYGRNYQVADIDASKLTHLNYA<br>FADICWNGKHGNPSTHPDNPNKQTWNCKESGVPLQNKEVPNGTLVLGEPWADVTKSYPGSGTTWEDCD<br>KYARCGNFGELKRLKAKYPHLKTIISVGGWTWSNRFSDMAADEKTRKVFAESTVAFLRAYGFDGVDLD<br>WEYPGVETIPGGSYRPEDKQNFTLLLQDVRNALNKAGAEDGKQYLLTIASGASQRYADHTELKKISQILD<br>WINIMTYDFHGGWEATSNHNAALYKDPNDPAANTNFYVDGAINVYTNEGVPVDKLVLGVPFYGRGWKS<br>CGKENNGQYQPCKPGSDGKLASKGTWDDYSTGDTGVYDYGDLAANYVNKNGFVRYWNDTAKVPYLY<br>NATTGTFISYDDNESMKYKTDYIKTKGLSGAMFWELSGDCRTSPKYSCSGPKLLDTLVKELLGGPINQKD<br>TEPPTNVKNIVVTNKNSNSVQLNWTASTDNVGVTEYEITAGEEKWSTTTNSITIKNLKPNTEYTFSIIAKDA<br>AGNKSQPTALTVKTDEANTTPPDGNGTATFSVTSNWGSGYNFSIIIKNNGTTPIKNWKLEFDYSGNLTQV<br>WDSKISSKTNNHYVITNAGWNGEIPSGGSITIGGAGTGNPAELLNAVISEN
